# Thin Cells of Polymer-Modified Liquid Crystals Described by Voronoi Diagrams

**DOI:** 10.3390/ma18051106

**Published:** 2025-02-28

**Authors:** Felicity Woolhouse, Ingo Dierking

**Affiliations:** Department of Physics and Astronomy, University of Manchester, Oxford Road, Manchester M13 9PL, UK; few29@cam.ac.uk

**Keywords:** Voronoi diagram, pattern formation, liquid crystal, PDLC, polymer stabilization, PSLC, polymer network

## Abstract

We investigated patterns formed during the polymerization process of bifunctional monomers in a liquid crystal for both large polymer concentrations (polymer-dispersed liquid crystals, PDLC) and small concentrations (polymer-stabilized liquid crystals, PSLC). The resulting experimental patterns are reminiscent of Voronoi diagrams, so a reverse Voronoi algorithm was developed that provides the seed locations of cells, thus allowing a computational reproduction of the experimental patterns. Several metrics were developed to quantify the commonality between the faithful experimental patterns and the idealized and generated ones. This led to descriptions of the experimental patterns with accuracies better than 90% and showed that the curvature or concavity of the cell edges was below 2%. Possible reasons for the discrepancies between the original and generated Voronoi diagrams are discussed. The introduced algorithm and quantification of the patterns could be transferred to many other experimental problems, for example, melting of thin polymer films, ultra-thin metal films, or bio-membranes. The discrepancies between the experimental and ideal Voronoi diagrams are quantified, which may be useful in the quality control of privacy windows, reflective displays, or smart glass.

## 1. Introduction

Self-organization is a field of increasing interest in physics, chemistry, material science and biology [1,2,3,4]. It describes the interdisciplinary field of statistically ordered structures, thus, pattern formation developed in non-equilibrium systems. General and well-known examples are spherulitic growth, rolls in cloud formation, Bernard convection cells, and oscillatory chemical reactions or growth patterns in bacteria colonies.

The non-equilibrium process investigated here is the phase separation of homogeneous liquid crystal and polymer mixtures via photopolymerization. By varying the ratios of liquid crystal and polymer or the domain sizes, the properties of the combined material can be tuned. Due to the response of liquid crystals under an applied voltage and their optical anisotropy, this results in a material that can be engineered both in preparation and as a device. This optically adjustable material has many applications for sustainable, efficient building design—crucial for a low energy future. Here, both polymer dispersed liquid crystals (PDLCs) and polymer stabilized liquid crystals (PSLCs) are considered.

**Figure 1:**
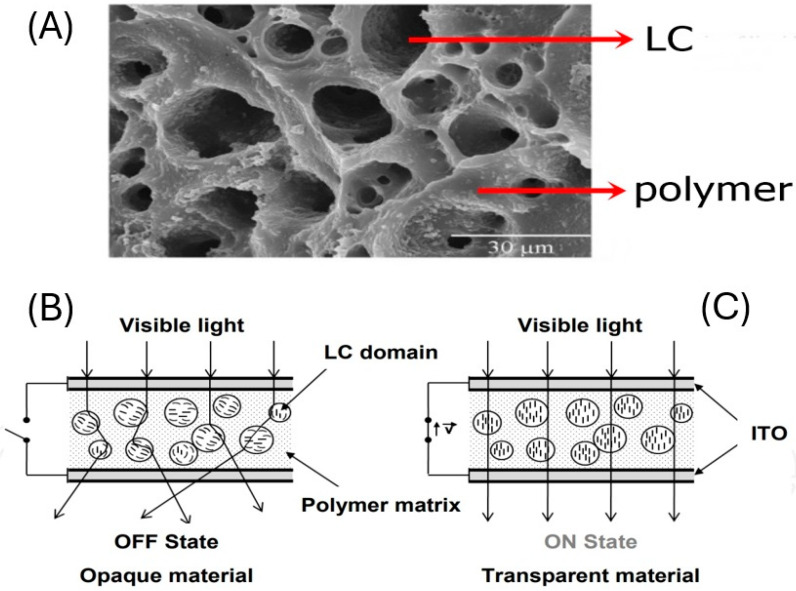
(**A**) Example of a PDLC structure with removed liquid crystal in the so-called Swiss cheese morphology. (**B**) Scattering off-state of the device, and (**C**) transparent on-state with index matching between ordinary refractive index of the liquid crystal and the polymer matrix. (Reproduced by permission after [10]).

PDLCs involve droplets of liquid crystals encapsulated within a polymer matrix [5]. The introduction of liquid crystals into the matrix phase causes the composite material to be opaque [6], due to light scattering based on the difference in refractive index between the liquid crystal and the matrix (see Figure 1). In order to align the optical axis of the liquid crystals, an electric field is applied. The refractive index along the long molecular axis, n_o_, is chosen such that it matches the refractive index of the polymer, which is called index-matching. In this configuration, called the on-state, the material becomes transparent [7]. After the electric field is removed, the liquid crystal returns to its original scattering configuration, called the off-state. The on- and off-states are used in commercial products for switchable windows, privacy windows and smart glass that can be “opened” and “closed” at will [8]. One advantage is the ability to regulate internal temperatures by the action of altering the window opacity [9]. PDLCs are generally formed at mixture compositions of about 40–60% polymer and exhibit a continuous polymer phase.

**Figure 2:**
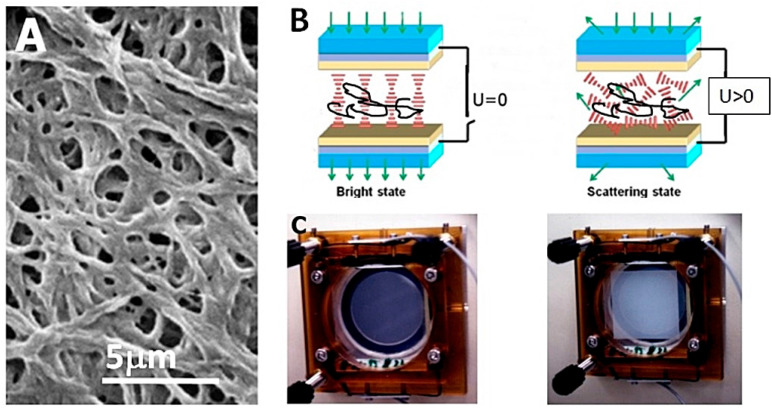
(**A**) Morphology of the polymer network with the liquid crystal removed. (**B**) At zero voltage, the polymerisation of bifunctional monomers is induced via UV radiation with a network forming that templates the LC orientation leading to a transparent off-state. Application of voltage causes breakup of the liquid crystal director field, which leads to the light scattering on-state. After field removal, the network elastically drives the LC back to its original state. (**C**) Some prototype devices from 1996 in the off- and on-state. (Reproduced by permission after [11]).

In contrast, PSLCs are formed at the opposite side of the phase diagram, at polymer concentrations clearly smaller than 10%, thus mainly consisting of liquid crystal and exhibiting a bi-continuous structure (see Figure 2). The polymer network templates the orientation of the liquid crystal and the device is transparent at zero applied voltage, i.e., transmissive in the off-state. Only when a voltage is applied above a certain threshold does the liquid crystal structure break up, inducing light scattering, which is the on-state. During subsequent removal of the field, the polymer network causes a rapid reorientation of the liquid crystal back to its original configuration, a process that could otherwise take hours or even days.

PDLC and PSLC samples can produce patterns that are closely described by a Voronoi diagram. From a state of uniformly distributed monomers, polymerization starts at different locations distributed statistically within the thin cell. At these locations the monomer concentration is depleted: by forming a polymer matrix in the case of PDLC, or a polymer network in the case of PSLC. This induces a non-equilibrium state with a concentration gradient, where monomer molecules migrate from regions of high concentration toward the initially formed polymer. These spots are the equivalent of Voronoi cell seeds, which are available via our algorithm. The radial movement of monomers along the concentration gradient is equivalent to the growth of circular regions. This process terminates when the monomers arrive at two forming polymer edges, which are then equivalent to the boundary edges of the individual Voronoi cells. As this happens throughout the material, a Voronoi pattern is formed.

Voronoi patterns or diagrams, which are also known as Dirichlet tessellations, are tilings of space that originate in a point, the seed, and grow radially outward until they encounter other cells and form a boundary. Voronoi diagrams date back to 1644 when Descartes published his Principia Philosophiæ to describe the distribution of matter in the universe. A mathematical foundation of Voronoi diagrams was laid by Dirichlet in the 19th century for two- and three-dimensional space [12]. Voronoi himself [13] then extended this description to general n-dimensional space.

Today, Voronoi diagrams find applicability in a wide variety of fields [14,15]. These range from astrophysics [16] to materials science [17,18,19,20] and medicine [21,22,23], particularly biology. Voronoi analysis finds its uses in the description of microstructure-dependent Young’s modulus and Poisson’s ratio [24], microplasticity of polycrystalline solids [25], or the elasticity of metal foams [26,27,28], as well as cracking analysis [29,30] and crack propagation [31]. In the life sciences, Voronoi cells are often used in the description and manufacturing of biomimetic structures, such as bone [32] or implants [33,34].

**Figure 3:**
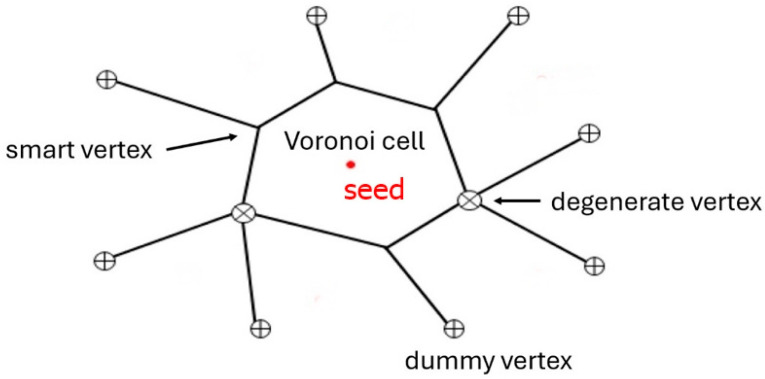
A basic representation of a Voronoi diagram in 2D, showing the boundaries as solid lines and the seed as a red point. The degenerate vertices are denoted with a crossed circle and the dummy vertices with a plus. Smart vertices are left as intersections of three cell edges.

A Voronoi diagram in the simplest sense is a method for tiling a space into a discrete set of cells that are each grown individually from a unique seed point. The growth rate of the cells is assumed to be constant across all seeds so that these are expanding radially. In 2D, every point either belongs to a cell and thus has a corresponding nearest seed, or it belongs to a boundary between two seeds. All cell boundaries are equidistant from their neighboring cell seeds, thus creating a perpendicular bisector of the line joining two adjacent seeds. Another property of a Voronoi diagram is that all vertex points created by intersecting boundaries are also equidistant from the seeds. This implies that the vertices are located at the center of a circle, with the seeds on the circumference. Vertices are distinguished into three categories, smart, degenerate and dummy vertices (Figure 3). Smart vertices are those in the bulk of the graph, with a degree of exactly 3. Degenerate vertices are bulk vertices with a degree greater than 3. Dummy vertices are not necessarily a vertex in the Voronoi sense but are created due to the finite size of the image.

The construction of a Voronoi diagram is straightforward, because one simply needs to grow circles simultaneously starting from different seeds and growing at constant speed. Once the boundaries of the circles meet, growth will cease, and the resulting structure shows a Voronoi diagram. An example can be found in [35]. The reverse process is far more complex, but it provides the physically important determination of the location of the seed points from an already formed Voronoi diagram. This marks the initiation point of the non-equilibrium process responsible for structure formation. Alternatively, the Voronoi description could be used to design specific polymer structures by strategically selecting initiation sites for polymerization or to predict the resulting structures in such scenarios. In this publication, we will apply a reverse Voronoi algorithm to the formation of PDLC and PSLC structures in thin sandwich cells.

## 2. Methodology

**Figure 4:**
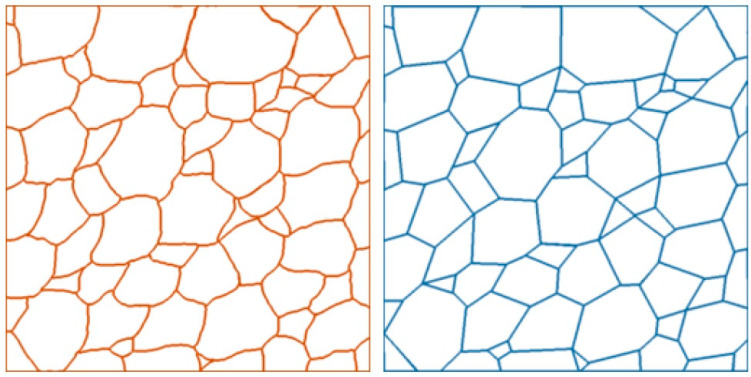
Comparison between faithful Voronoi-like pattern (**left**) and idealized Voronoi pattern (**right**) where vertices are inevitably connected by straight lines.

Before calculating the seed positions, the experimental microscopy images of both PDLC and PSLC needed to be pre-processed. Suitable sample areas were identified and square regions isolated to arrive at images that were compatible with our algorithm, ensuring that the images can be efficiently processed using standard computing. From these images, dummy, smart and degenerate vertices were identified and localized via ImageJ 1.54 [36]. Joining the vertices with straight lines gave an idealized image of the Voronoi pattern. A faithful pattern reproducing the concave and convex edges was also taken to be able to compare the real experimental structure with the idealized Voronoi one, as depicted in Figure 4.

There have been many attempts to find an efficient and accurate solution to the inverse Voronoi problem in the last 40 years [37,38,39]. The algorithm we used to generate the seeds of the potential Voronoi pattern was developed as an inverse Voronoi generator for liquid crystal textures [40,41]. An abridged summary of the algorithm used is presented below.

**Figure 5:**
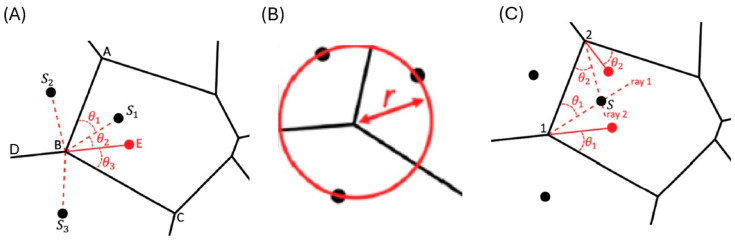
Schematic figures to illustrate the geometric conditions available for the calculation of seed points from a given Voronoi pattern. (**A**) Cell boundaries are mirror planes to seeds of neighboring cells. (**B**) Seeds of adjacent cells lie on a circle centred on their common vertex. (**C**) Voronoi construction with dummy vertexes.

Following Schoenberg [42], the algorithm is based on the geometric properties of Voronoi patterns: (i) any boundary between two cells is the perpendicular bisector at the midpoint of a line joining the two seeds of those cells. This means that there exists reflective symmetry of two seeds in two adjacent cells on their cell boundary (see Figure 5A). (ii) Seeds of adjacent cells lie on a circle centred on their common vertex (see Figure 5B). (iii) In relation to a dummy vertex D and its rotated point R, it can be shown that the angles θ_2_ and θ_1_ are equal, thus θ_2_ = θ_1_ (see Figure 5C). If a pattern only contains one smart vertex, the above conditions cannot be used to determine the seed point. However, a Voronoi cell with only one smart vertex always has two neighbouring cells. In this case one can use condition (iii) with the single smart vertex and a ray can be constructed that goes through the seed. Condition (ii) can then be employed to generate the seed point from knowledge of the seeds of the neighbouring cells. If a pattern contains no smart vertex at all, (iii) cannot be used and mirroring is the only possibility to proceed. One has to proceed in a similar way for cells with only degenerate vertices.

**Figure 6:**
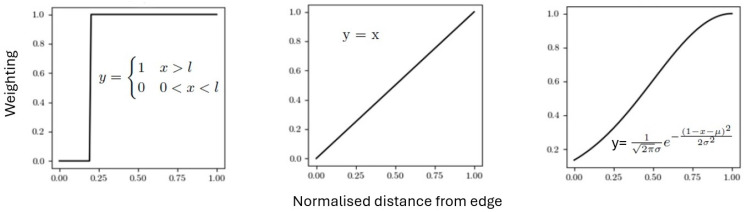
Gradient functions employed and respective equations: step (**left**), linear (**middle**), and Gaussian (**right**). In each case x is the normalised distance from the cell edge of the pixel. x = 0 corresponds to an edge pixel, and x = 1 to the centre.

With the above algorithm one can compute the seeds of all cells. To allow a more quantitative comparison, we attributed different weighting approaches to the borders of the cells. As each pixel can only belong to the cell corresponding to the nearest seed, finding the closest seed to each pixel returns the cell each pixel belongs to. Every pixel was weighted by its distance from the cell border and a gradient function. We used several gradient functions (Figure 6), which in a sense account for cell borders of finite size and human judgement when constructing an idealized Voronoi pattern.

We note that for all studies carried out, the step function was by far the best model to simulate the borders. This outcome was determined by considering how much each gradient was removed from the sample and how well it matched the borders. The aim was to retain as many pixels as possible. From visual comparisons with the sample, it was seen that all gradients except the single pixel step model removed too many of the pixels, without a justifiable increase in sample replication.

**Figure 7:**
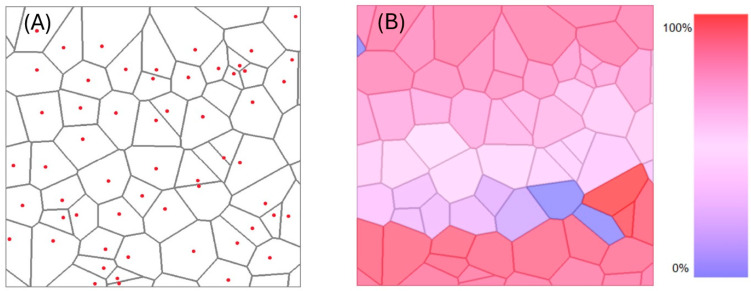
(**A**) Exemplary result of a calculated Voronoi pattern, providing the seed points of each cell (some are outside of the picture). (**B**) Comparison with the original Voronoi pattern via a (relative) heat map derived from the Sørensen–Dice coefficient. Red represents areas of highest matching and blue represents areas of lowest matching.

Once we established the reverse Voronoi algorithm and a decision on how best to represent the edges of the cells in a Voronoi pattern, we could arrive at a generated representation of the pattern, including the positions of the seeds for each cell. A commonality between the original and the calculated pattern could be calculated using a Sørensen–Dice statistic to calculate a coefficient C_SD_ for each cell. This could then be assigned to a colour scheme based on its relative value to the other cells of the diagram to produce a heat map that visualised the areas of high and low accuracy between the two Voronoi patterns. This is depicted in Figure 7. We note that the heat maps depicted in this study were deliberately all individually normalized to the range 0–100%, to emphasize that differences in local deviations can be visualized. This largely over-emphasizes the deviations and does not imply that red and blue describe perfect or no correlation. In fact, in this investigation the deviations were very small. The heat maps simply enlarge this relatively small scale to the range of 0–100% and thus do not allow a comparison between different samples. By choosing an absolute scale, the latter can obviously be achieved, if so desired.

**Figure 8:**
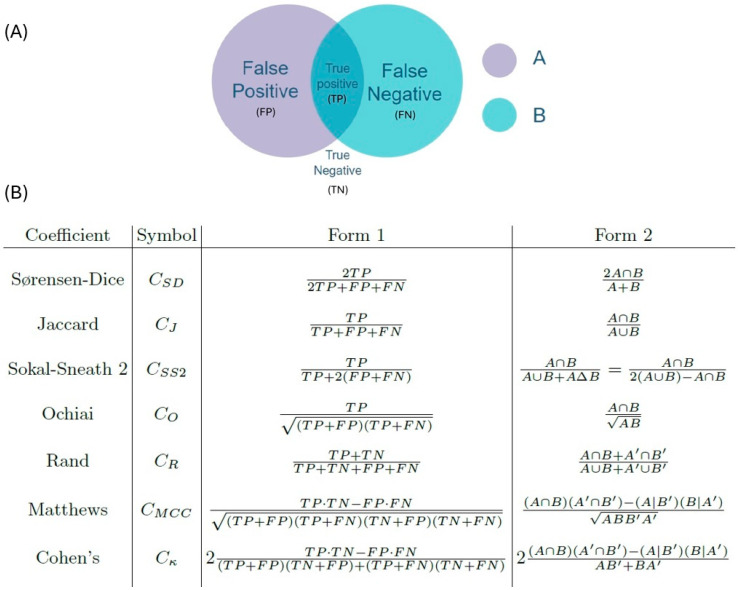
(**A**) Venn diagram of the relationship between pixel intersections and classification. (**B**) Definitions of the different commonality coefficients that can be used to compare sets A and B. Form 1 is the classification form and Form 2 the probability form.

In formulating a useful similarity metric, several coefficients were considered, including the Rand index [43], the Jaccard coefficient [44], the Sørensen–Dice coefficient [45,46], the Matthews correlation coefficient [47], Cohen’s Kappa [48], the Ochiai measure [49] and Sokal–Sneath similarity 2 [50]. The classic measure of accuracy is the Rand coefficient [51]; when C_R_ is corrected for chance agreement, it is identical to C_SD_ [52]. To find a good metric for the data, one must first consider the type of data that is being analysed. If there are two populations being compared, with every element either having a characteristic or not, then the data can be described as dichotomous, quantitative, or qualitative [53]. Dichotomous data have + 1 if the characteristic is present and −1 if the characteristic is absent. Qualitative data have +1 if the characteristic is shared, whilst quantitative data take the range [0, 1] for the similarity between two elements. The data type considered in this case was qualitative data, as each pixel is either shared between two cells or it is not. When considering the intersection of the two populations, the pixels could be categorised as true negatives (TN), true positives (TP), false positives (FP) and false negatives (FN) (see Figure 8). The relationship between the two models is as follows: if two pixels are in both images A and B then they are TP; if two pixels are in neither A or B then they are TN. Also, if a pixel from A is not in B then it is FP, if a pixel from B is not in A then it is FN.

The considered coefficients and equations are shown in Figure 8. In the comparison function, each cell in image A had its pixels compared with those of the same cell in image B. As by definition the pixels in the considered cell are in either image A or B, there were never any pixels considered that were in neither A or B. There is thus no information on TN pixels, which implies that coefficients requiring the number of TN can be excluded from further consideration, leaving only C_SD_, C_J_, C_O_, and C_SS2_. The Rand index, Matthews correlation coefficient, and Cohen’s Kappa were thus not considered further.

The most prevalent uses of these coefficients are artificial intelligence image segmentation and biological genetic comparisons. Their use depends upon available data and the type of classification required. For manufacturing problems, C_SD_, C_J_, and C_SS2_ perform the best [54]. In ecological studies C_SD_ and C_J_ are the most commonly used [55,56]. Both C_SD_ and C_J_ are used to produce dendrograms [57]. One drawback of using C_SD_ and C_J_ is that in situations of small sample size they are negatively biased [58]. The coefficient used here is based upon the C_SD_ coefficient, which is equal to twice the intersection divided by the total of the two populations. This was modified by adding the intersection separately weighted by population A and population B, divided by the totals, which were also weighted. This modified C_SD_ coefficient was used to compare the models and to compute the final score for the accuracy of the Voronoi descriptions of the individual experimental samples.

Before coming to the actual results on experimental PDLC and PSLC sample patterns, we would like to consider the question of comparability a bit further, as this is an important issue to judge the quality of the procedure and algorithm. Also, the illustrated methodology can most likely be applied in a range of other different systems, such as the melting of thin polymer films or ultra-thin metal films, and possibly also in biological membrane systems.

As a next step one can determine how much the faithful image deviates from the idealised image (as depicted in Figure 4). This was quantified by the difference in C_SD_ score between the generated and idealised, *C_GI_*, and generated and faithful, *C_GF_*, images:

(1) $$D=\left|C_{GI}-C_{GF}\right|.$$

The larger the magnitude of *D* in Equation (1), the more concavity the original sample displays. Furthermore, the *C_IF_* value between the idealised and faithful images sets an upper theoretical limit for the similarity between the generated Voronoi pattern and the faithful image, *C_GF_*. Normalising *C_GF_* with the upper limit, *C_IF_,* resulted in a measure for the fit of the generated image to the faithful whilst accounting for the limit set, by removing the curvature. This measure is denoted *M*:

(2) $$M=\frac{C_{GF}}{C_{IF}}.$$

The *C_GI_* score, additionally, measures how accurate the generated image is, whilst also removing concavity. The difference in these two scores is a measure of the errors introduced by the method used to account for concavity. This can be imagined as a path difference; *M* compares the generated image to the sample then accounts for concavity, whilst *C_GI_* accounts for concavity then compares the generated image. By calculating the difference between the two measures, the discrepancy between the two scores is given as:

(3) $$∆=\left|M-C_{GI}\right|.$$

A smaller Δ indicates that the generated Voronoi diagram fits the original sample more closely, as there is less discrepancy caused by the difference of the generated image to the faithful or idealised images. This was determined both visually and by calculating the relative errors for both metrics, using Equation (3), resulting in

(4) $$d=\frac{∆}{M}\;\;\;\;\;\;\;and\;\;\;\;\;d\prime =\frac{∆}{C_{GI}}.$$

A visual comparison was made between the faithful and generated images and the faithful and idealised images. Superimposing the images showed those with smaller discrepancies, quantified by Δ. Equation (4) gives the errors of *M* and *C_GI_*, respectively.

## 3. Results and Discussion

Having discussed the methodology and procedure in quite some detail and having developed a methodology to quantify the accuracy of the determined reverse-engineered Voronoi pattern, we can now proceed to demonstrate the actual results on PDLC and PSCT patterns.

The experimental images for the polymer dispersed liquid crystal (PDLC) samples were taken from the literature [59]. The sample was composed of 80% liquid crystal E7 (a eutectic mixture of biphenyls, 4-cyano-n-pentyl-biphenyl (5CB), 4-cyano-n-heptyl-biphenyl (7CB), 4-cyano-n-octyloxy-biphenyl (8CB), and 4-cyano-n-pentyl-p-terphenyl), approximately 10% HDDA (hexamethylene diacrylate) cross-linker, around 1% DMPAP (2-Dimethylamino-4-(methyl-phenylamino)-phenol) photo-initiator, and 10% polymer TMHA, a polymer that contains 3,5,5-trimethylhexyl acrylate, for samples PDLC-1 and PDLC-3, or polymer A3DA, an acrylate monomer that is used to create polymer dispersed liquid crystal (PDLC) films, for sample PSLC-2 [59]. We note that with about 20% polymer material, the PDLC was on the low side of polymer content for PDLCs, yet its electrooptic performance was indeed quite impressive [59].

**Figure 9:**
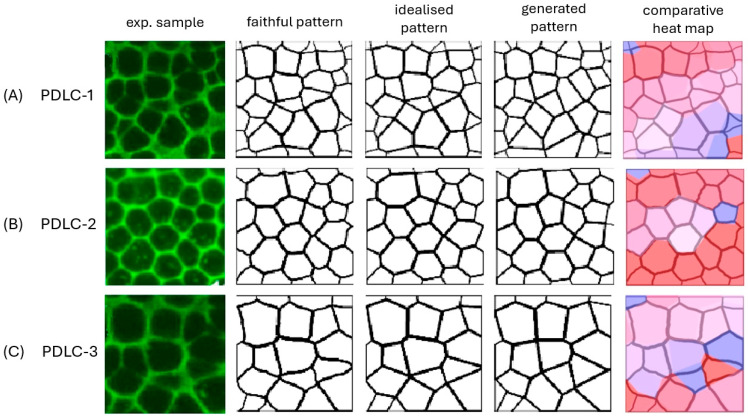
Experimental sample [59], faithful pattern, idealised representation, generated Voronoi pattern and comparative heat map for polymer dispersed liquid crystal samples (**A**) PDLC-1, (**B**) PDLC-2, and (**C**) PDLC-3.

Sample PDLC-3 was a subsection of PDLC-1 with less cells, in order to investigate the effect of cropping the sample image. The preparation method used was PIPS (polymerization-induced phase separation). Figure 9 depicts the sample images, together with their faithful representation, idealised presentation, generated Voronoi pattern and heat map to identify how well regions of cells were reproduced.

Already, one can visually see that the patterns observed in the PDLC samples were very well described by the generated Voronoi pattern, albeit with some differences in structure. The harshest measure of the accuracy is the *C_GF_* score, which is the Sørensen–Dice score between the generated and the faithful pattern. The overall accuracy of representation between generated and faithful patterns was (87.8 ± 1.4)%, which was much decreased for PDLC-1 with 81% as compared to the cropped image PDLC-3 with 92%. This in turn implies that images with less cells accumulate a smaller error and thus better accuracy in Voronoi re-generation. This is of course understandable, as each cell contributes to the overall deviation between experimental original and calculated patterns.

**Figure 10:**
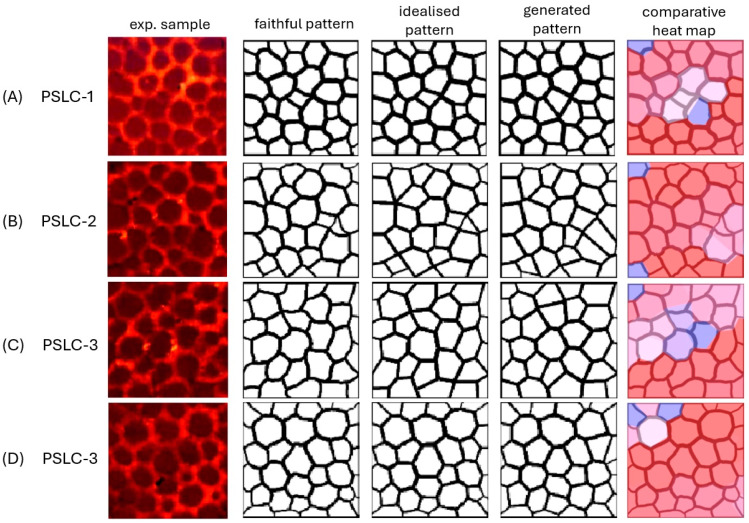
Experimental sample, faithful pattern, idealised representation, generated Voronoi pattern and comparative heat map for polymer stabilised liquid crystal samples (**A**) PSLC-1, (**B**) PSLC-2, (**C**) PSLC-3, and (**D**) PSLC-4.

The polymer stabilised liquid crystal (PSLC) samples PSLC-1 to PSLC-4 were composed of 95 wt% liquid crystal 5CB (4-Cyano-4′-pentylbiphenyl) and 5 wt% RM257 monomers, polymerised by UV irradiation for 90 min with the addition of a small amount of photo-initiator BME (benzoin methyl ether). Figure 10 illustrates the sample images, together with their faithful representation, idealised presentation, generated Voronoi pattern and heat map.

Qualitatively, the results of the PSLCs were very similar to those of the PDLCs. The experimental structure of the polymer network was well described by a Voronoi pattern, with the commonality between generated and faithful (experimental) pattern averaging (92.3 ± 0.3)%. The heat maps reveal only small areas where the coincidence between experiment and calculation deviated.

In fact, one could argue that it is surprising that the accordance between experiment and theory is so high. The reason lies in several aspects: (i) the start of polymerization is a process of a statistical nature, while for the calculation of Voronoi patterns it is always assumed that all seeds start growing at the same time. (ii) A constant growth rate cannot necessarily be assumed for the experiment, as it is for the calculation. (iii) Curvature in the cell boundaries is observable in the experimental images, while the simulation of Voronoi patterns can only lead to straight edges. (iv) The experimental cell boundaries are of finite thickness, while the calculated ones are ideally infinitesimally narrow, or here, one pixel thick. Nevertheless, the step-like gradient function did provide the best results, as already stated above. Given these principal limitations on any Voronoi description of experimental patterns, the correspondence between the two patterns with a commonality of approximately 90% observed for the PDLC as well as the PSLC structures is impressive. The complete data set for the investigated samples is provided in Table 1.

On examination of the results for *D*, the concavity of the samples was typically less than 2%, with a mean of <*D*> = (1.0 ± 0.7)% for the PDLC system and <*D*> = (0.8 ± 0.2)% for the PSLC system. This means that concavity is small and can in first approximation be neglected, which is also indicated by the Sørensen–Dice score between the idealised and the faithful pattern, *C_IF_*, which in all cases was not far below 100%.

**Figure 11:**
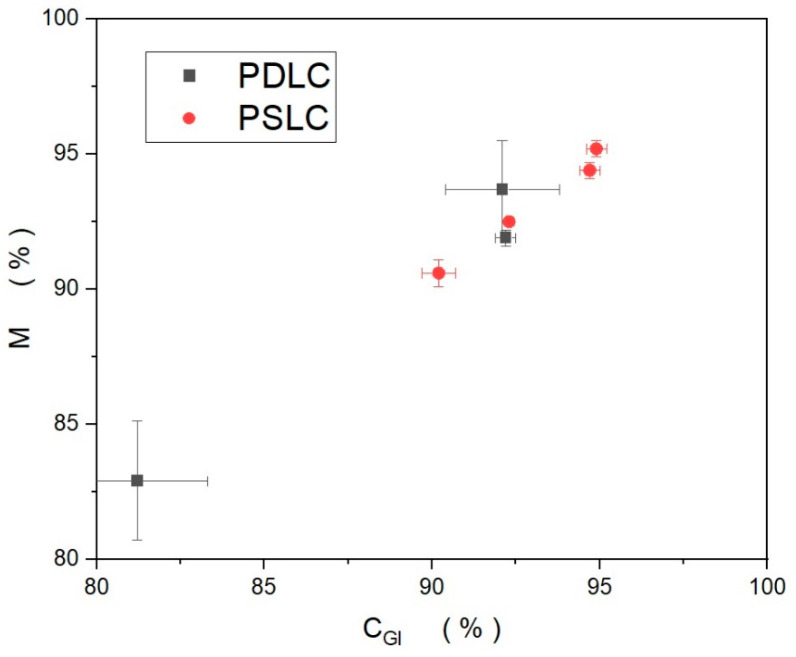
Comparison between *C_GI_* and *M* for both PDLC (black squares) and PSLC (red circles) samples.

We recall that *C_GI_* is the Sørensen–Dice score between the generated and idealized images, where in the idealized image the edge curvature of Voronoi cells is removed. *M* is the ratio between the scores for the generated vs. faithful and the idealized vs. faithful pattern, Equation (2). Thus, the closer *M* is to 100%, the smaller the difference between faithful and idealized image patterns, i.e., the less the curvature observed in the boundaries of the experimental Voronoi cells. Figure 11 shows a comparison of *M* vs. *C_GI_* for both PDLCs and PSLCs, with errors given by *d* and *d*’ respectively. The results for both sets scaled proportionally, with a slope close to 1. This is further confirmation that the concavity of the Voronoi edges is small and did not contribute significantly to the description via the reverse algorithm. A similar conclusion can be drawn from Δ. A smaller Δ indicates that the generated Voronoi diagram fits the original sample more closely, as there is less discrepancy caused by the difference between the generated image and the faithful or idealised images. A source of uncertainty, the concavity, was successfully removed, seen by comparing *M* and *C_GF_*. As *M* gives a consistently higher accuracy than *C_GF_*, the removal of the curvature via an intermediate idealised image worked effectively, showing that the step function is a suitable model for the cell boundaries.

Further, the heat maps could be used to analyse the experimental procedure used. They can highlight potential areas of deformation in sample preparation via areas that do not conform to a Voronoi diagram as accurately. This analysis can thus be used to feed back to experimental techniques. One consideration would be to standardise the heat maps, which would allow for inter-sample comparison. This may be a useful technique to use when preparing and analysing multiple samples with the same equipment, for example in quality control.

The presented reverse Voronoi algorithm and the detailed discussion of how to evaluate its accuracy and commonality between calculated and experimentally observed patterns could be extended to other fields of research, for example, the melting of thin polymer films, ultra-thin metal films, membrane structures and the like.

## 4. Conclusions

We developed an algorithm that describes polymer patterns of polymer-modified liquid crystals obtained in thin sandwich cells as Voronoi diagrams. For both polymer-dispersed liquid crystals (PDLC) and polymer-stabilized liquid crystals (PSLC), similar results were obtained. From experimentally determined patterns, the reverse Voronoi algorithm determined the seed positions and cell edges to an accuracy of generally better than 90%. Several different commonality scores were introduced based on the Sørensen–Dice coefficient to compare the faithful original experimental pattern to the idealized and generated patterns, which remove possible curvature in the boundary edges of Voronoi cells. This is best performed via a step function to describe the boundaries, and it was shown that the concavity of the boundaries lay below 2% and could in first approximation be neglected. The produced heat maps illustrate sample regions where the Voronoi description was not as accurate than for other regions, which is also of importance for applications, for example, quality control in the production of PDLC-based privacy windows, reflective displays or smart glass. The developed methodology can be applied to further systems, such as the melting of thin polymer films, ultrathin metal films, or biological membranes, just to name a few possible further applications. Alternatively, one could utilize the description to predict pre-determined polymer structures through simulation of polymerization sites.

## Figures and Tables

**Table 1:** First three columns of scores are produced by the algorithm, with *C_IF_* being the Sørensen–Dice score between idealised and faithful patterns, *C_GF_* that between the generated and faithful patterns, and *C_GI_* that between the idealised and generated Voronoi patterns. Other values are calculated from these scores according to Equations (1)–(4). All accuracies are given as percentage.

Sample	Number of Cells	*C_IF_*	*C_GF_*	*C_GI_*	*D*	*M*	Δ	*d*	*d*′
PDLC-1	37	97.6	81.0	81.2	0.21	82.9	1.74	2.10	2.15
PDLC-2	30	98.8	92.5	92.1	0.48	93.7	1.62	1.73	1.76
PDLC-3	22	97.7	89.8	92.2	2.39	91.9	0.31	0.34	0.34
PSLC-1	34	99.0	94.3	94.9	0.66	95.2	0.29	0.31	0.31
PSLC-2	33	98.1	89.0	90.2	1.25	90.6	0.43	0.48	0.48
PSLC-3	30	99.1	93.6	94.7	1.15	94.4	0.30	0.32	0.32
PSLC-4	36	99.6	92.1	92.3	0.27	92.5	0.11	0.12	0.12

## Data Availability

The original contributions presented in this study are included in the article. Further inquiries can be directed to the corresponding author.
